# Supplementary material for: Human serum triglycerides promote Staphylococcus aureus biofilm formation and antibiotic tolerance
Source: NPJ Biofilms Microbiomes. 2026 Jun 21;12:188. doi: 10.1038/s41522-026-01064-x (PMC13631273; doi:10.1038/s41522-026-01064-x)
Supplement: Supplementary file 1 [file 41522_2026_1064_MOESM1_ESM.pdf]

Human serum triglycerides promote *Staphylococcus aureus* biofilm formation and antibiotic tolerance

Elizabeth V. K. Ledger[1,2#], Niamh E. Horgan[1,2], Denis Lynch[3], Lorraine M. Bateman[3,4] and Ruth C. Massey[1,2,5]

[1]School of Microbiology, University College Cork, Cork, Ireland.

[2]APC Microbiome Ireland, University College Cork, Cork, Ireland.

[3]School of Chemistry, Analytical and Biological Chemistry Research Facility, University College Cork, Cork, Ireland.

[4]School of Pharmacy, University College Cork, Cork, Ireland.

[5]School of Cellular and Molecular Medicine, University of Bristol, Bristol, UK.

# for correspondence: eledger@ucc.ie

Supplementary figures 1-8

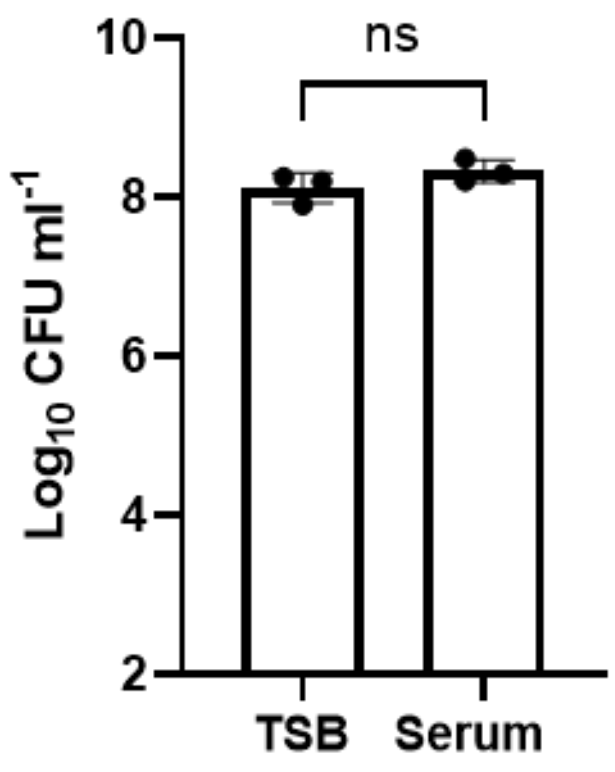

Fig. S1. Bacterial numbers do not increase during incubation in serum. There was no significant difference in bacterial survival as measured by $\log_{10}$ CFU ml^{-1} of the JE2 WT strain after growth in TSB and after the 16 h incubation in serum. Data represent the geometric mean ± geometric standard deviation of three independent repeats and were analysed by t-test (ns, $p > 0.05$).

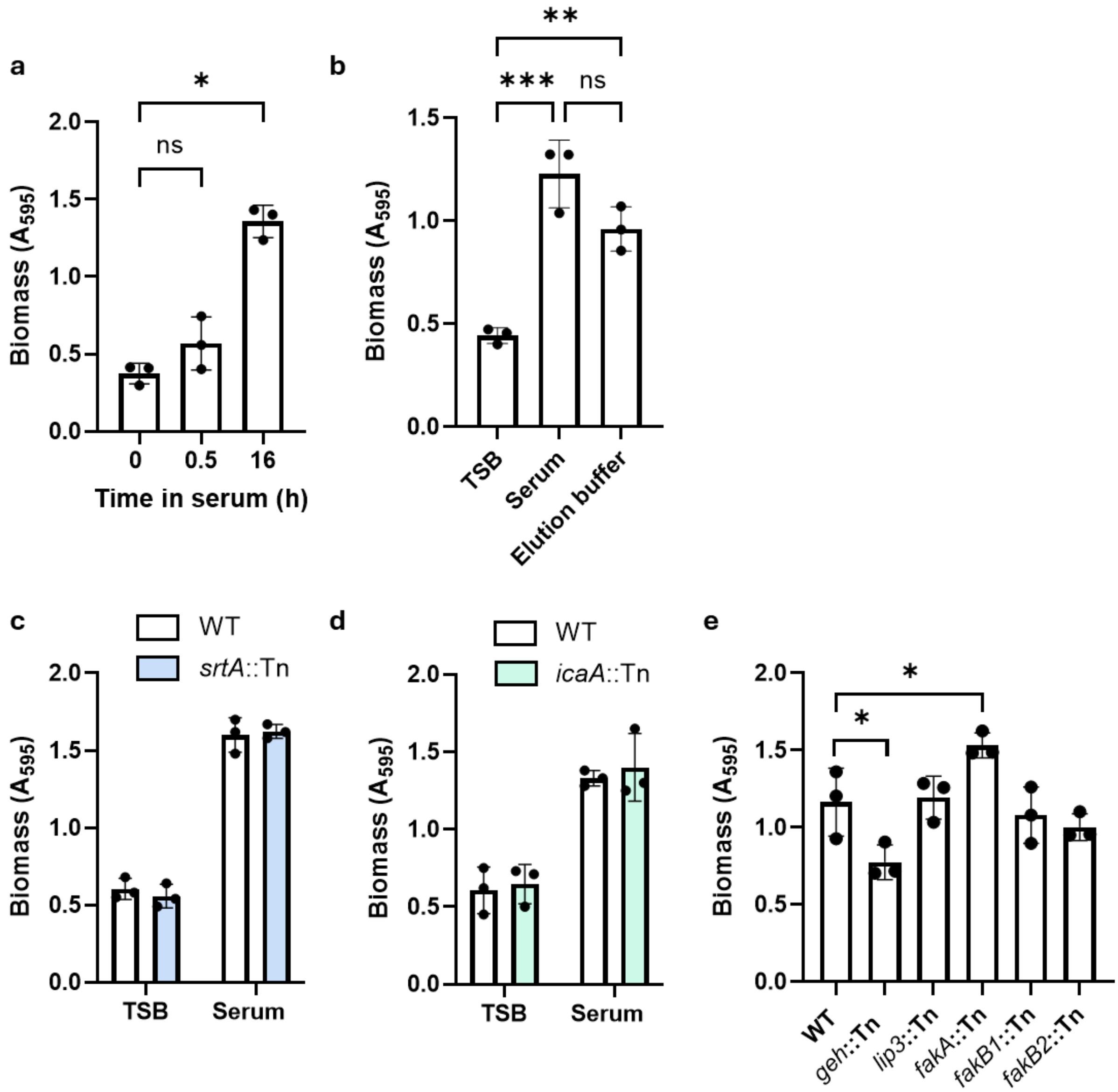

Fig. S2. Increased biofilm formation in serum is not fully explained by host factors sticking to *S. aureus* and does not require *srtA, icaA* or fatty acid kinase. (a) JE2 WT was grown in TSB and then incubated in serum for 0, 0.5 or 16 h before being inoculated into TSB and incubated for 24 h to form biofilm before quantification of biofilm biomass with crystal violet. (b) XXX. The JE2 WT strain and (c) the *srtA*::Tn mutant, (d) the *icaA*::Tn mutant and (e) the *geh*::Tn, *lip3*::Tn, *fakA*::Tn, *fakB1*::Tn and *fakB2*::Tn mutants were TSB-grown and serum-incubated before being inoculated into TSB and incubated for 24 h to form biofilm, which was then quantified using crystal violet. Data represent the mean ± standard deviation of three independent biological repeats. Each biological repeat is the mean of three technical replicates. Data in a and b were analysed by one-way ANOVA with Sidak's *post-hoc* test. ns, $P > 0.05$, *, $P < 0.05$, **, $P < 0.01$ ***, $P < 0.001$. Data in c and d were analysed by two-way ANOVA with Sidak's *post-hoc* test, for all WT vs mutant comparisons, $P > 0.05$. Data in e were analysed by one-way ANOVA with Dunnett's *post-hoc* test. *, $P < 0.05$.

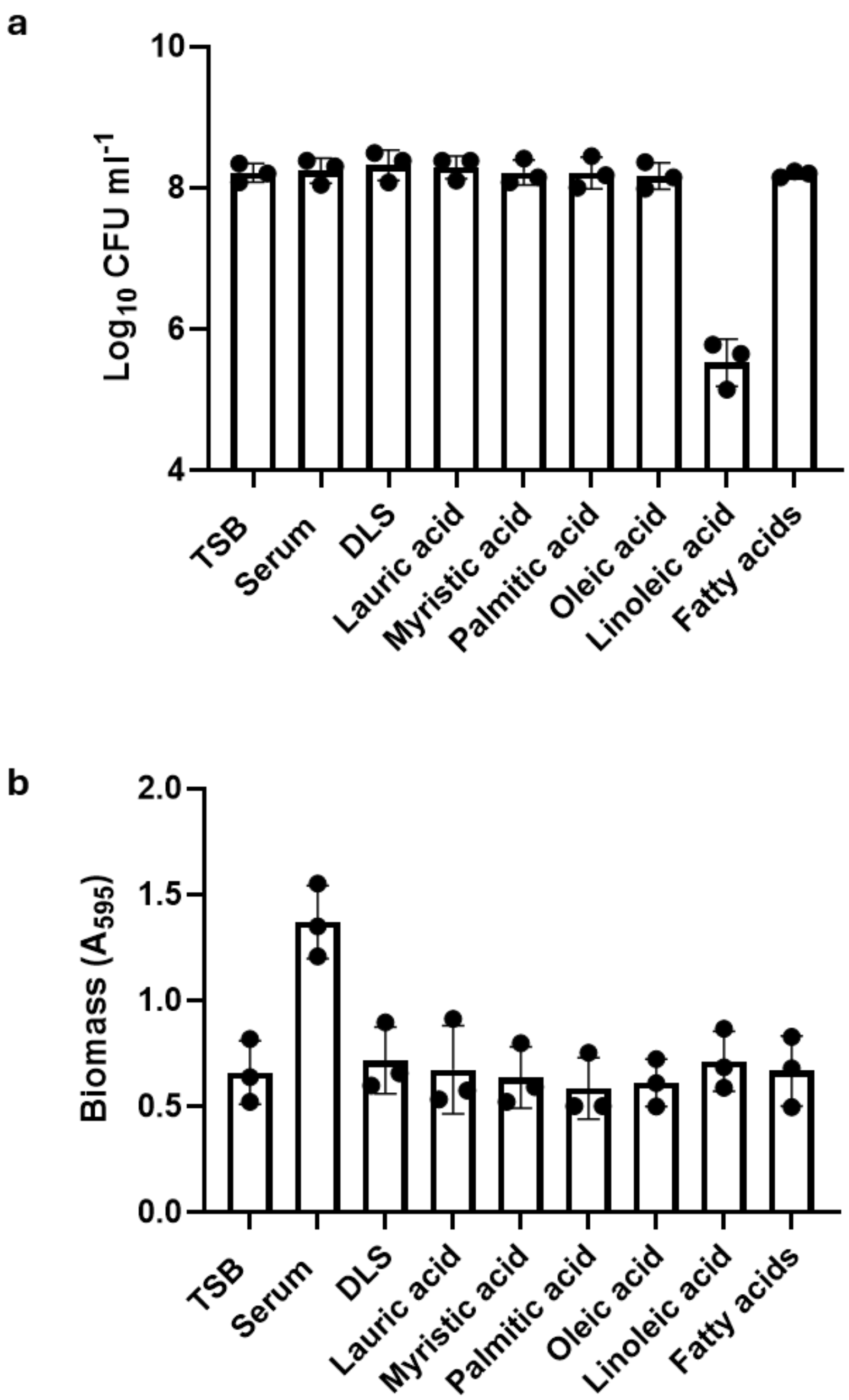

Fig. S3. Addition of fatty acids does not promote biofilm formation. JE2 WT was TSB-grown, serum-incubated or incubated in DLS supplemented or not with 1 mM individual fatty acids or 5 mM total fatty acids before (a) $\log_{10}$ CFU ml^{-1} were determined and (b) bacteria were inoculated into TSB, incubated for 24 h to form biofilm and biofilm quantified with crystal violet. Data represent the mean ± standard deviation of three independent repeats. Data were analysed by one-way ANOVA with Dunnett's *post-hoc* test. In panel a, there were no significant differences between bacterial survival in DLS and any other condition except linoleic acid (*, $P < 0.05$). In panel b, there was no significant difference between biomass in DLS or DLS supplemented with any fatty acid.

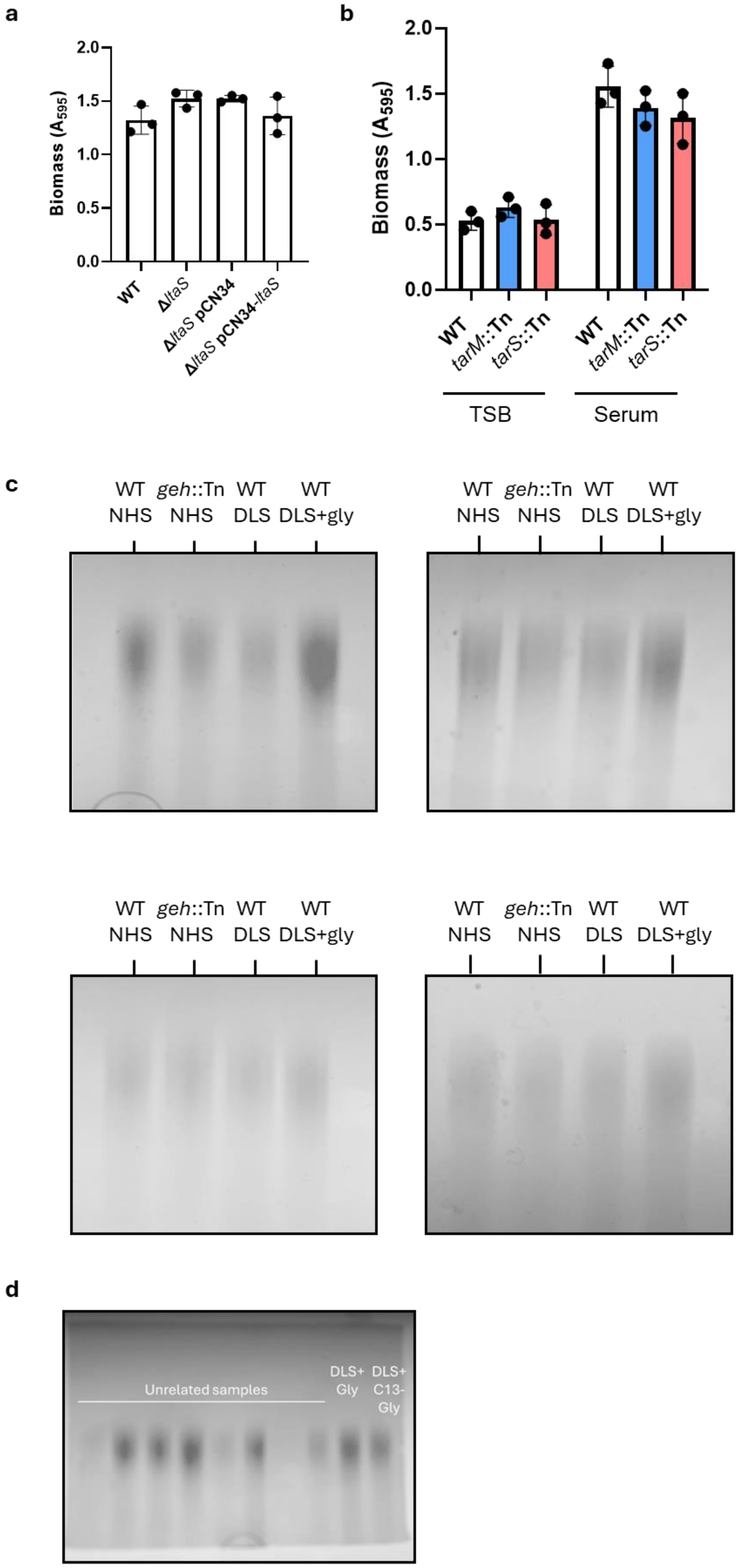
a
Biomass (A595)
2.0
1.5
1.0
0.5
0.0
WT
ΔltaS
ΔltaS pCN34
ΔltaS pCN34-ltaS
b
Biomass (A595)
2.0
1.5
1.0
0.5
0.0
WT
tarM::Tn
tarS::Tn
WT
tarM::Tn
tarS::Tn
TSB
Serum
c
WT NHS
geh::Tn NHS
WT DLS
WT DLS+gly
WT NHS
geh::Tn NHS
WT DLS
WT DLS+gly
WT NHS
geh::Tn NHS
WT DLS
WT DLS+gly
WT NHS
geh::Tn NHS
WT DLS
WT DLS+gly
d
Unrelated samples
DLS+ Gly
DLS+ C13-Gly

Fig. S4. LTA synthesis and WTA glycosylation are not required for biofilm formation, but increased WTA content is associated with serum-induced biofilm formation. (a) Biomass of biofilms formed from serum-incubated cultures of LAC* WT, the Δ*ltaS*-S2 mutant, the Δ*ltaS*-S2 mutant carrying the empty pCN34 plasmid or the Δ*ltaS*-S2 mutant complemented with *pCN34-ltaS* as measured by crystal violet staining. (b) The JE2 WT strain and the *tarM*::Tn and *tarS*::Tn mutants were TSB-grown and serum-incubated before being inoculated into TSB and incubated for 24 h to form biofilm, which was then quantified using crystal violet. (c) Native PAGE analysis of WTA extracted from JE2 WT and the *geh*::Tn mutant after incubation in NHS, DLS or DLS supplemented with glycerol. Each gel represents an independent biological replicate. These gels were quantified using ImageJ to generate the graph in Fig. 3f. (d) Uncropped gel from Fig. 3g. NHS, normal human serum; DLS, delipidated serum; gly, glycerol. Data in a and b represent the mean ± standard deviation of three independent biological repeats where each repeat is the mean of three technical replicates. Data in a were analysed by one-way ANOVA with Dunnett's *post-hoc* test and data in b were analysed by two-way ANOVA with Sidak's *post-hoc* test. For all comparisons (WT vs mutants under each condition), $P > 0.05$.

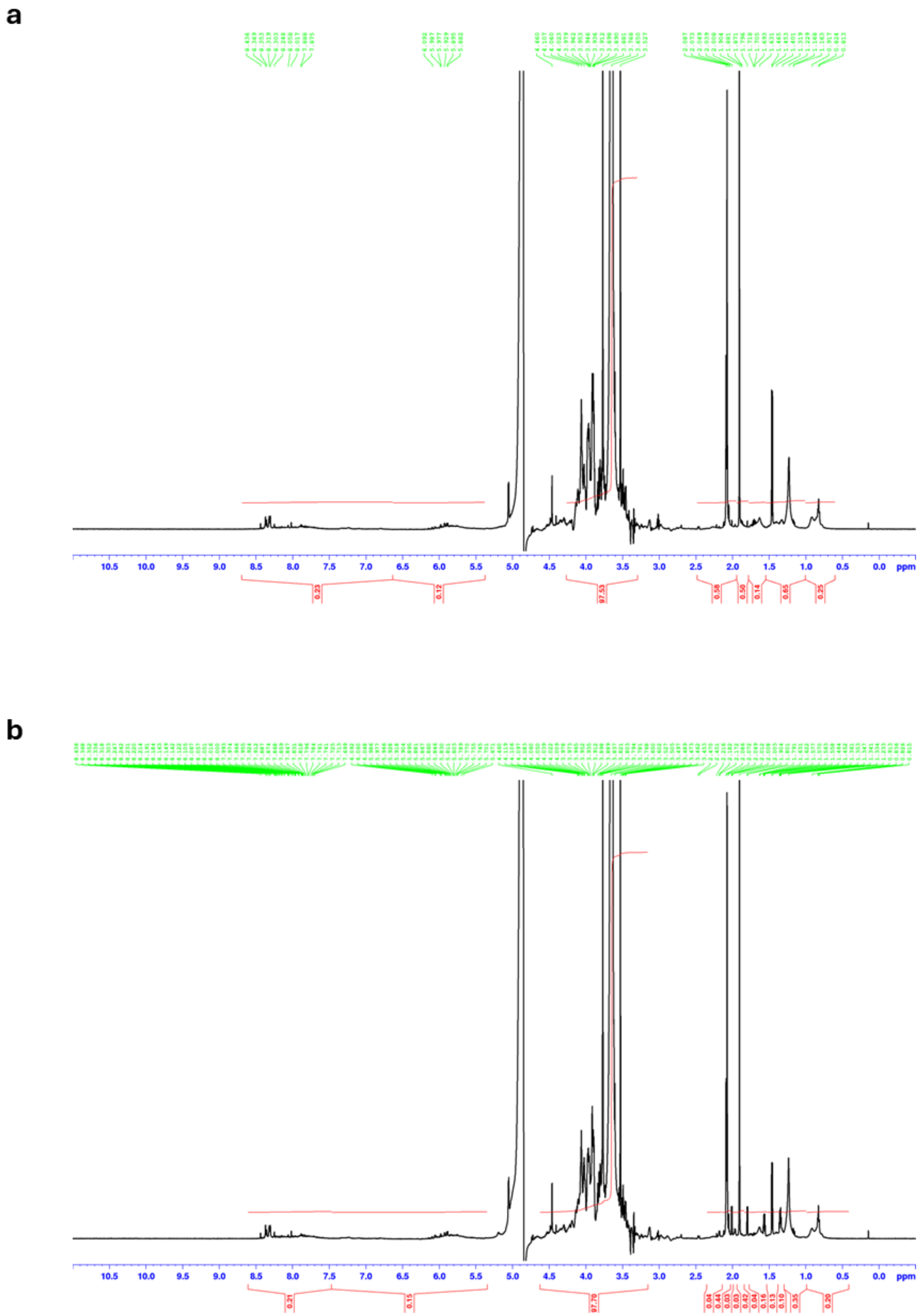

Fig. S5. ^{1}H NMR spectra. ^{1}H NMR (water suppression, 90:10 H_2O/D_2O, 600 MHz) spectrum of (a) DLS + glycerol WTA extraction (in 0.2M tris HCl) and (b) DLS + ^{13}C-glycerol WTA extraction (in 0.2 M tris HCl).

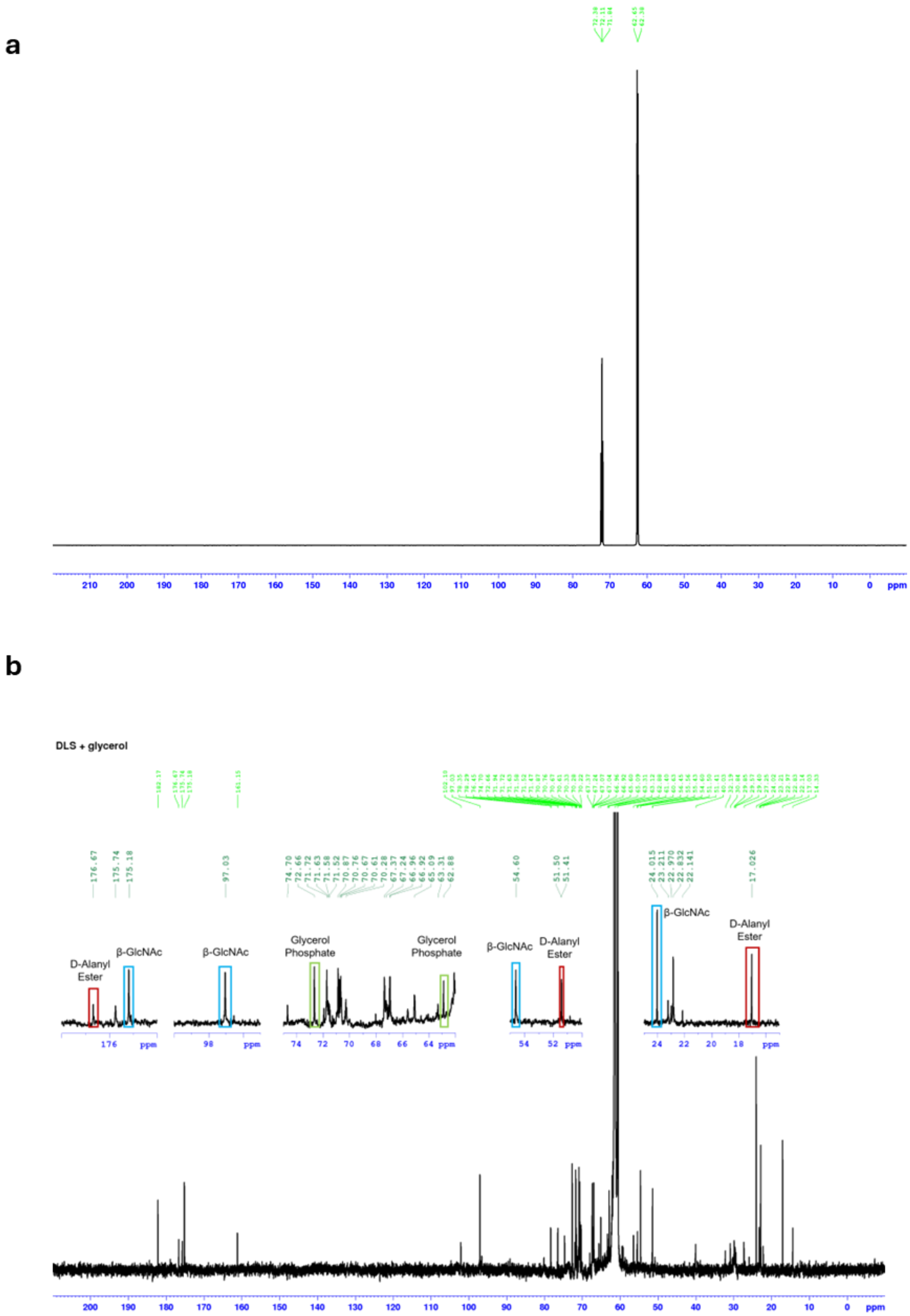
a
b
DLS + glycerol
176.67
175.74
175.18
97.03
54.60
51.50
51.41
24.015
23.211
22.970
22.832
22.141
17.026
D-Alanyl Ester
β-GlcNAc
β-GlcNAc
Glycerol Phosphate
Glycerol Phosphate
β-GlcNAc
D-Alanyl Ester
β-GlcNAc
D-Alanyl Ester
ppm

c

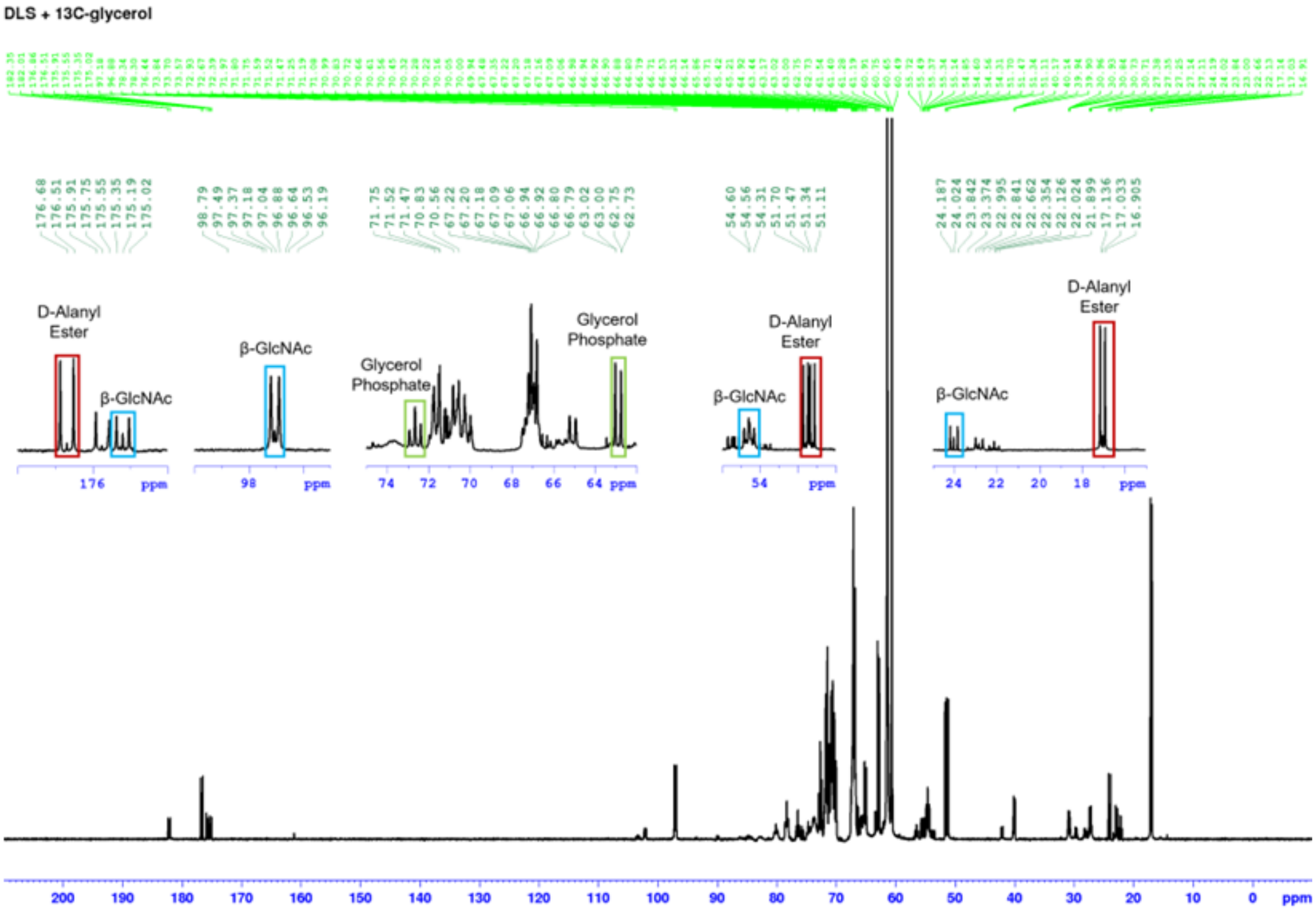

d

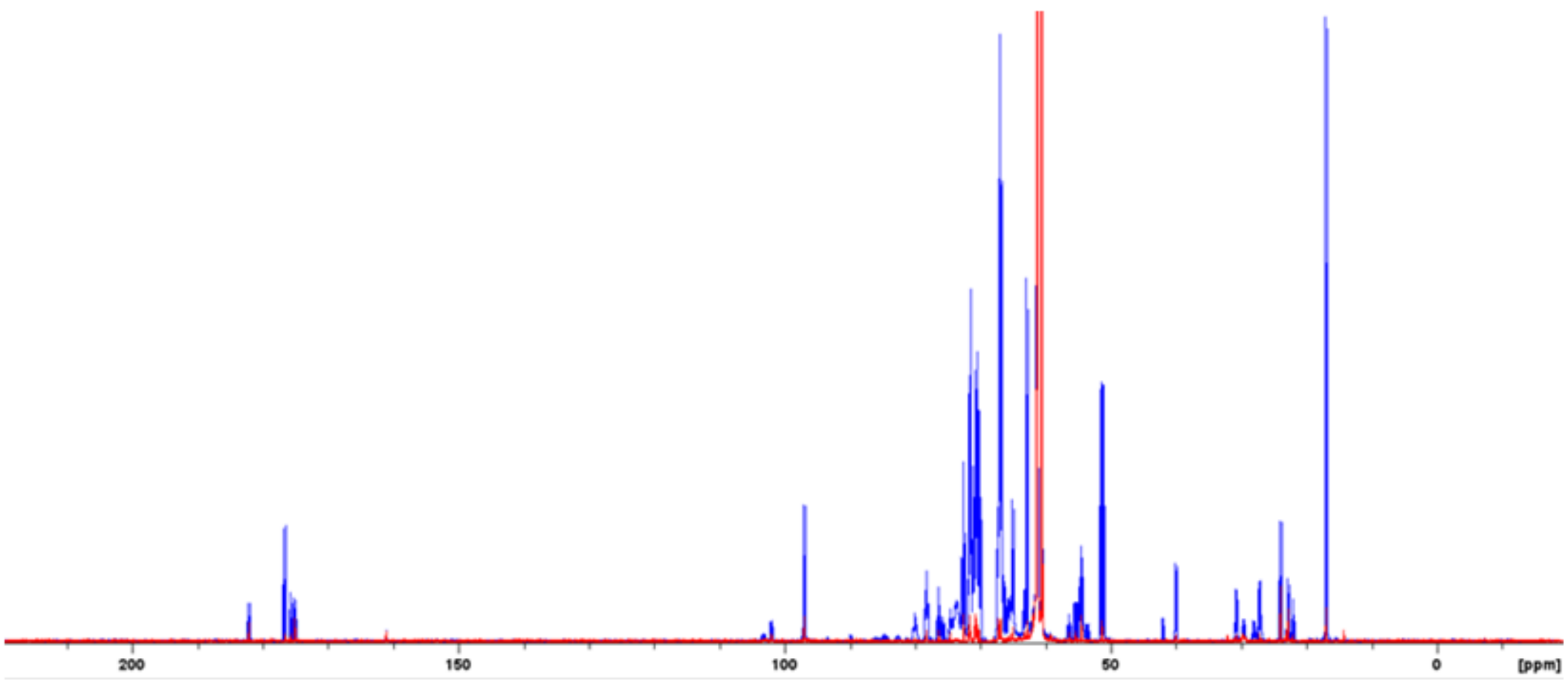

Fig. S6. ^{13}C NMR spectra. (a) ^{13}C NMR (UDEFT, 90:10 H_2O/D_2O, 150.9 MHz) spectrum of ^{13}C-glycerol. (b) ^{13}C NMR (UDEFT, 90:10 H_2O/D_2O, 150.9 MHz) spectrum of DLS + glycerol sample (WTA extraction in 0.2 M tris HCl). (c) ^{13}C NMR (UDEFT, 90:10 H_2O/D_2O, 150.9 MHz) spectrum of DLS + ^{13}C-glycerol sample (WTA extraction in 0.2 M tris HCl). (d) Directly overlain ^{13}C NMR (UDEFT, 90:10 H_2O/D_2O, 150.9 MHz) spectra of DLS + ^{13}C-glycerol sample (WTA extraction in 0.2 M tris HCl) (blue) and DLS + C-glycerol sample (WTA extraction in 0.2 M tris HCl) (red). The tris signals are scaled to the same intensity for both spectra shown.

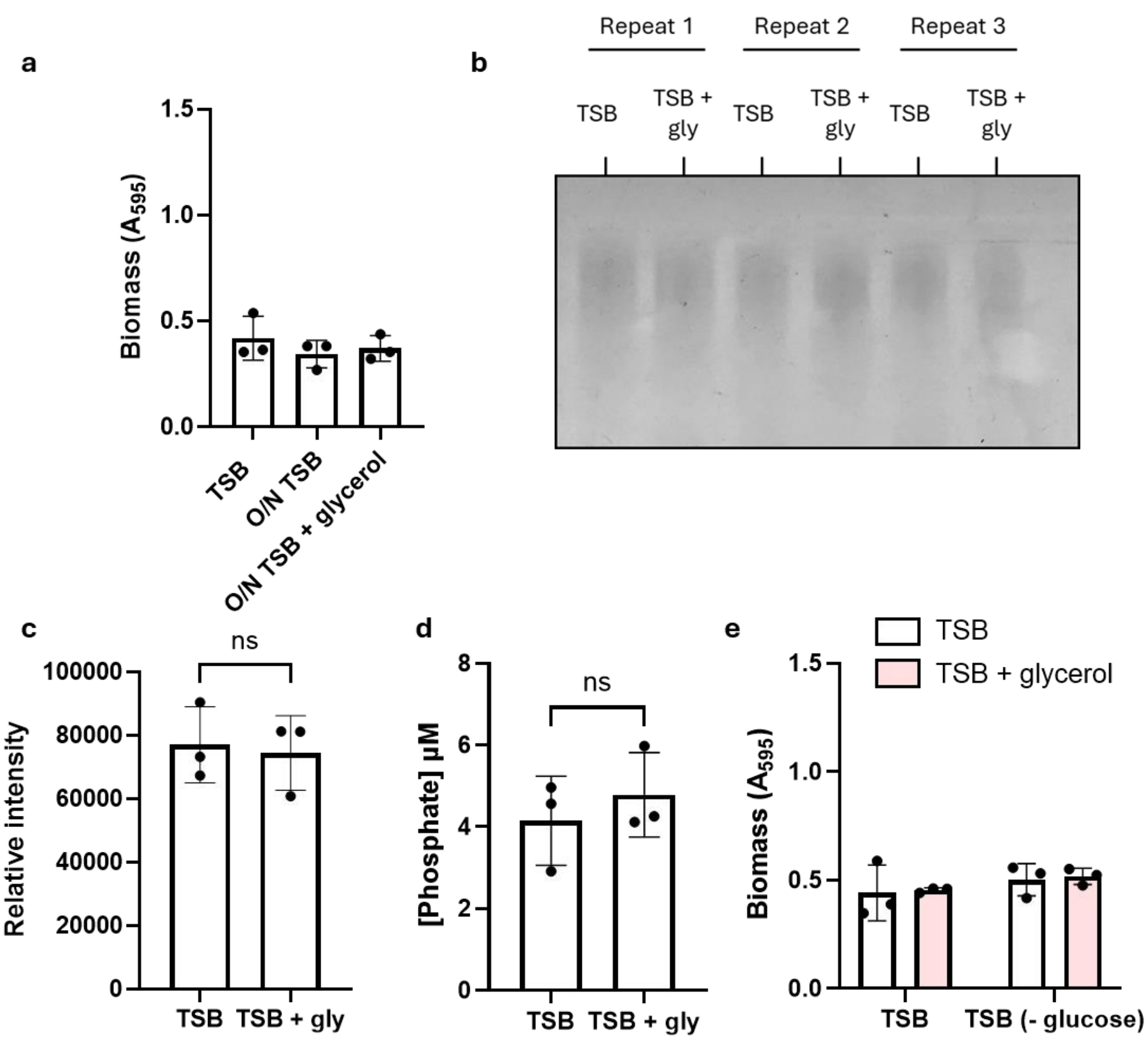

Fig. S7. Addition of glycerol into TSB does not promote biofilm formation. JE2 WT grown to mid-exponential phase in TSB, grown for 16 h in TSB or for 16 h in TSB supplemented with 0.1% glycerol before (a) being inoculated into TSB and subsequent biofilm formation measured by crystal violet and (b) WTA were extracted and analysed by native PAGE and alcian blue staining. WTA were quantified by (c) measuring the intensity of the alcian blue staining using ImageJ and (d) measuring phosphate concentration using a colorimetric assay. (e) JE2 WT was grown for 16 h in TSB or in TSB without glucose supplemented, or not, with 0.1% glycerol before being inoculated into TSB and subsequent biofilm formation measured by crystal violet staining. Data represent the mean ± standard deviation of three independent biological repeats. In a and e, each biological repeat is the mean of three technical replicates. Data in a were analysed by one-way ANOVA with Tukey's *post-hoc* test (for all comparisons, $P > 0.05$). Data in c and d were analysed by t-test and data in e were analysed by two-way ANOVA with an uncorrected Fisher's LSD.

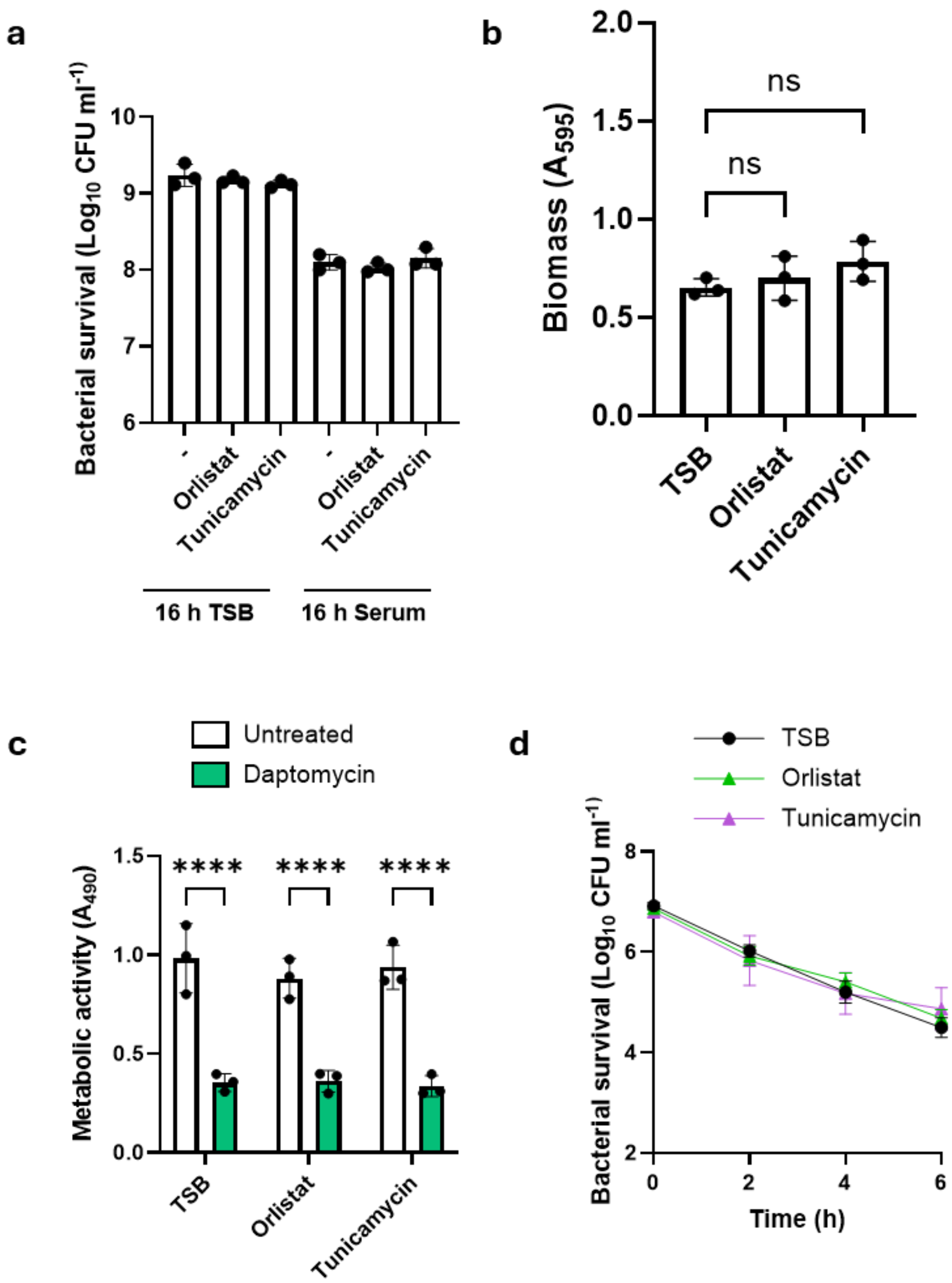

Fig. S8. (a) Log_{10} CFU ml^{-1} counts of JE2 WT after incubation in TSB or serum supplemented, or not, with orlistat (100 µg ml^{-1}) or tunicamycin (0.5 µg ml^{-1} in TSB or 128 µg ml^{-1} in serum; this difference is to account for the increased concentration required in serum due to inactivation by serum protein binding). (b) Biofilm formation as measured by crystal violet staining of JE2 WT grown in TSB or TSB supplemented with orlistat (100 µg ml^{-1}) or tunicamycin (0.5 µg ml^{-1}). (c) Metabolic activity of biofilms formed from JE2 WT after being incubated for 16 h in TSB supplemented or not with 100 µg ml^{-1} orlistat or 0.5 µg ml^{-1} tunicamycin and then exposed, or not, to 20 µg ml^{-1} daptomycin for 24 h as measured by XTT. (d) Biofilms formed from JE2 WT which had been incubated in TSB supplemented or not with 100 µg ml^{-1} orlistat or 0.5 µg ml^{-1} tunicamycin were disrupted

and exposed to 20 μg ml^{-1} daptomycin for 6 h and log_{10} CFU ml^{-1} determined every 2 h. Data in a and b were analysed by one-way ANOVA with Dunnett's *post-hoc* test (for all untreated vs treated comparisons, $P > 0.05$). Data in c were analysed by two-way ANOVA with Tukey's *post-hoc* test (****, $P < 0.0001$) and data in d were analysed by two-way ANOVA with Dunnett's *post-hoc* test (for all untreated vs treated comparisons, $P > 0.05$).
